# Editorial: Celebrating Microbial Diversity: The Many Cell Cycles of Eukaryotic Microbes

**DOI:** 10.3389/fcimb.2021.738994

**Published:** 2021-07-28

**Authors:** Catherine J. Merrick, Sabrina Absalon, Mathieu Brochet, Ziyin Li, Elena S. Suvorova

**Affiliations:** ^1^Department of Pathology, Cambridge University, Cambridge, United Kingdom; ^2^Department of Pharmacology and Toxicology, School of Medicine, Indiana University Bloomington, Indianapolis, IN, United States; ^3^Faculty of Medicine, University of Geneva, Geneva, Switzerland; ^4^Department of Microbiology and Molecular Genetics, McGovern Medical School, University of Texas Health Science Center at Houston, Houston, TX, United States; ^5^Department of Internal Medicine, Division of Infectious Diseases and International Medicine, Morsani College of Medicine, University of South Florida, Tampa, FL, United States

**Keywords:** cell cycle, replication, parasite, *Plasmodium*, *Toxoplasma*, *Trypanosoma*

The cell cycle is a fundamental process in biology: all living organisms must be able to generate new cells. The ways in which they do this are surprisingly variable. Cell cycle machinery has been studied in exquisite detail over several decades in model organisms such as bacteria, unicellular yeasts and mammalian cells, giving rise to the concept of ‘The cell cycle’ – a binary division process that is stereotypic and highly conserved. However, as horizons have expanded beyond these model organisms, it has become clear that binary division is not the only mode of replication. In particular, early-diverging eukaryotic microbes often divide by ‘unconventional’ means, such as schizogony in malaria parasites and endodyogeny in *Toxoplasma* parasites. In fact, microbial cell cycles are so diverse as to raise the question: ‘Does a ‘conventional’ cell cycle actually exist?’ Non-binary-fission cycles are common among protozoan parasites, including most of the Apicomplexa, a phylum including medically important parasites such as *Plasmodium, Babesia, Toxoplasma* and *Cryptosporidium* (Striepen et al., 2007), and they are also found in very different organisms such as atypical fungi (Gladfelter, 2006).

This Research Topic explores the breadth of eukaryotic cell cycles at the cellular and molecular levels through Reviews, Perspectives and Primary Research articles. These articles report upon the diverse cell cycles of eukaryotic protozoan parasites: their molecular mechanisms and the concepts that unify or divide these organisms.

Two review articles explore the modes of cell division in Apicomplexan parasites. Gubbels et al. discuss the concept of plasticity – how the same organism can vary its mode of cell division and the number of progeny it produces at different points in its parasitic life cycle. This is a common theme in Apicomplexa, which may, for example, switch between endodyogeny and schizogony by altering the circuitry that couples or uncouples genome replication, karyokinesis and cytokinesis. In binary fission, these processes are hardwired to occur in succession, whereas a fundamental concept of more flexible, ‘unconventional’ cell cycles is that they can be uncoupled. Morano and Dvorin, meanwhile, focus on one part of the molecular machinery that is crucial for generating daughter cells, the basal complex. How does this contractile ring, which separates new cells, differ in Apicomplexa compared to model organisms such as *S. cerevisiae*, and what can this tell us about how these divergent organisms achieve cell division?

Continuing the focus on Apicomplexa, two perspective articles discuss some of the most conceptually challenging aspects of schizogony in *Plasmodium*. Simon et al. ask ‘How many is enough?’ – how do parasites measure and control the number of progeny generated by syncytial division, and how might this have evolved to maximise parasite fitness in variable host environments? A complementary article from Machado et al. discusses the issue of how a parasite undergoing schizogony copes with cellular genome contents from 1n to >20n, asking if there are unique implications for managing transcription in these unusual syncytial cells.

Finally, two primary research articles examine some very different parasites, the kinetoplastids *Trypanosoma cruzi* and *Trypanosoma brucei*. In these, division is ‘conventionally’ binary, but the executive machinery is very divergent. In particular, it seems that the whole eukaryotic kingdom has evolved only one way to divide a replicated genome: a cell must assemble a mitotic spindle and pull the sister chromatids apart using microtubules. But how is that mitotic spindle built? There is certainly more than one way to generate a centriole or a kinetochore, just as Morano and Dvorin suggest that there may be more than one way to build a contractile ring. Brusini et al. report a proteomic study of the *T. brucei* kinetochore, which contains almost none of the proteins used in yeast or human kinetochores. Alonso et al. examine the tubulin component of microtubules in *T. cruzi*, elucidating the role of acetylation in regulating these microtubules.

Overall, this collection of articles ranges from the conceptual to the highly molecular – accurately reflecting the state of the art in research on diverse eukaryotic cell cycles. Beyond the basic biological interest of this topic, it is also highly important to global One Health because many eukaryotic microbes are major parasites of humans and animals, causing diseases such as malaria, toxoplasmosis, avian coccidiosis and African sleeping sickness. Targeting the unusual cell cycles of such parasites is a potential avenue for chemotherapy that would be parasite-specific, leaving the cell biology of mammalian or avian hosts unaffected. Accordingly, research interest is rising rapidly, particularly in the cell cycles of Apicomplexa, and this has coincided with major advances in the availability of research tools and molecular-genetic technologies (Shen et al., 2014; Merrick, 2015; Tandel et al., 2019). Nevertheless, our knowledge of these fascinating organisms is still limited by the development of tools and *in vitro* culture systems. Parasites such as *Cryptosporidium*, *Theileria* and *Sarcocystis* are less accessible to research and therefore remain relatively understudied, as does an enormous variety of uncultured fungal species (Mitchison-Field et al., 2019). A lot of fascinating research remains to be done before we can claim to understand the full breadth of eukaryotic cell cycles.

## Author Contributions 

CM wrote the manuscript. All authors contributed to the article and approved the submitted version.

## Funding

CM: ERC research grant ‘Plasmocycle’. ZL: NIH R01 grant AI101437. MB: Swiss National Science Foundation 31003A_179321.

## Conflict of Interest

The authors declare that the research was conducted in the absence of any commercial or financial relationships that could be construed as a potential conflict of interest.

## Publisher’s Note

All claims expressed in this article are solely those of the authors and do not necessarily represent those of their affiliated organizations, or those of the publisher, the editors and the reviewers. Any product that may be evaluated in this article, or claim that may be made by its manufacturer, is not guaranteed or endorsed by the publisher.
